# Culture-Confirmed Multilobar Cavitary Pulmonary Tuberculosis Despite a Negative QuantiFERON-TB Gold Assay in an Immunocompetent Young Adult

**DOI:** 10.7759/cureus.112415

**Published:** 2026-07-10

**Authors:** Irina Ter-Ovanesyan, Claudia Pedreira, Kian Memari, Anastasiya Sizova, Carlos Ramirez-Matos, Shane Williams, Peter Cohen, Lissette P Lazo

**Affiliations:** 1 Medical School, Nova Southeastern University Dr. Kiran C. Patel College Of Osteopathic Medicine, Fort Lauderdale, USA; 2 Family Medicine, Nova Southeastern University Dr. Kiran C. Patel College Of Osteopathic Medicine, Fort Lauderdale, USA; 3 Family Medicine, Palmetto General Hospital, Hialeah, USA

**Keywords:** acid-fast bacilli, atypical tuberculosis, cavitary lung disease, interferon-gamma release assay, multilobar cavitation, pulmonary tuberculosis, quantiferon false negative

## Abstract

Pulmonary tuberculosis (TB) remains a major global health concern and may present with radiographic and laboratory findings that complicate timely diagnosis. Interferon-gamma release assays (IGRAs), including QuantiFERON-TB Gold, are blood tests that measure T-cell immune sensitization to *Mycobacterium tuberculosis* antigens; they do not distinguish latent infection from active disease and cannot exclude active TB. We report a 23-year-old immunocompetent Venezuelan-born man who presented with fever, night sweats, productive cough, and intermittent hemoptysis after approximately seven months of respiratory symptoms. Chest imaging demonstrated multilobar cavitary disease involving both upper lobes and the left lower lobe. Despite a negative QuantiFERON-TB Gold result, two sputum acid-fast bacilli smears were positive, *M. tuberculosis* nucleic acid amplification testing was positive, and mycobacterial culture confirmed the *M.* *tuberculosis* complex. The patient was placed in airborne isolation and treated with rifampin, isoniazid, pyrazinamide, and ethambutol. Public health authorities coordinated directly observed therapy, contact investigation, and outpatient sputum monitoring. This case demonstrates that a negative IGRA cannot rule out active pulmonary TB and that atypical multilobar cavitation should not lower clinical suspicion when epidemiologic, clinical, and microbiologic findings support the diagnosis.

## Introduction

Tuberculosis (TB) remains one of the leading infectious causes of illness and death worldwide. The WHO estimated that approximately 10.7 million people developed TB in 2024 [1]. In the United States, 10,388 TB cases were reported in 2024, corresponding to an incidence of 3.1 cases per 100,000 persons [2]. United States TB-control activities include timely diagnosis, airborne isolation, contact investigation, treatment of latent infection, and completion of treatment using patient-centered support such as directly observed therapy (DOT), which the Centers for Disease Control and Prevention (CDC) considers the standard of care for TB disease [2,3].

Pulmonary TB classically presents with chronic cough, fever, night sweats, weight loss, and hemoptysis. A pulmonary cavity is an air-containing space formed within an area of diseased lung, usually as a consequence of tissue necrosis. Post-primary TB commonly produces cavities in the apical and posterior upper lobe segments, where higher oxygen tension favors mycobacterial growth and cavitation is associated with increased organism burden and transmission [4]. Nevertheless, multilobar involvement, nodular consolidation, and lower-lobe cavitation may occur and can resemble necrotizing bacterial pneumonia, fungal infection, septic emboli, vasculitis, or malignancy [4,5].

An IGRA is a blood test that measures interferon-gamma released by sensitized T lymphocytes after exposure to TB-specific antigens. Interferon-gamma release assays (IGRAs) are useful for identifying immune sensitization to *Mycobacterium tuberculosis*, particularly in the evaluation of latent infection, but they cannot distinguish latent infection from active disease and should not be used to exclude active TB [6]. False-negative results occur in culture-confirmed disease, including in patients without recognized major immunosuppression [7,8]. When active pulmonary TB is suspected, evaluation should proceed with acid-fast bacilli (AFB) smear microscopy, nucleic acid amplification testing (NAAT), and mycobacterial culture regardless of the IGRA result [6,9].

We describe an immunocompetent young adult with culture-confirmed pulmonary TB despite a negative QuantiFERON-TB Gold assay and an atypical multilobar cavitary pattern. The case emphasizes the need to prioritize clinical probability and microbiologic confirmation over a single negative immune-based test.

## Case presentation

A 23-year-old Hispanic man originally from Venezuela, who had immigrated to the United States six years earlier, presented to the emergency department with one week of fever, chills, night sweats, sore throat, and productive cough. He also reported a waxing and waning cough for approximately seven months, with intermittent hemoptysis, fatigue, and other constitutional symptoms. He denied recent travel, incarceration, homelessness, alcohol use disorder, tobacco use, malignancy, chronic corticosteroid use, biologic therapy, or known human immunodeficiency virus (HIV) infection. He initially denied known TB exposure but later recalled that a close family member had previously been treated for TB. He did not recall prior TB testing, and his bacille Calmette-Guérin vaccination status was unknown.

On presentation, his temperature was 38.6°C, heart rate was 112 beats/minute, and oxygen saturation was 97% on room air. Examination showed oropharyngeal erythema and coarse bilateral breath sounds. No cervical, supraclavicular, axillary, or inguinal lymphadenopathy was appreciated. He denied bone pain, joint swelling, back pain, abdominal pain, hyperpigmentation, dizziness, or symptoms suggestive of adrenal involvement.

Initial testing demonstrated a normal total white blood cell count with relative neutrophilia, relative lymphopenia, mild microcytic anemia, mild thrombocytosis, substantially elevated inflammatory markers, mild hypoalbuminemia, and hyperproteinemia (Table 1). The erythrocyte sedimentation rate was 72 mm/hour, more than three times the upper reference limit, and the C-reactive protein concentration was 8.6 mg/dL, approximately 8.6 times the upper reference limit. The estimated absolute lymphocyte count, calculated from the total leukocyte count and lymphocyte percentage, was approximately 0.95 × 10³/µL, indicating mild lymphopenia. The total protein-albumin difference was 5.9 g/dL, consistent with an increased globulin fraction in the setting of chronic inflammation, although this finding was nonspecific. HIV antigen/antibody testing was nonreactive. A multiplex respiratory viral polymerase chain reaction panel was negative for influenza A and B, respiratory syncytial virus, severe acute respiratory syndrome coronavirus 2, and the other viruses included in the local panel. Point-of-care rapid group A streptococcal antigen testing was negative. QuantiFERON-TB Gold testing returned negative.

**Table 1:** Initial laboratory and infectious screening results. *Calculated as total white blood cell count multiplied by the lymphocyte fraction. HIV: human immunodeficiency virus; TB: tuberculosis; g/dL: grams per deciliter; µL: microliter; fL: femtoliter; mm/hour: millimeters per hour; mg/dL: milligrams per deciliter; U/L: units per liter.

Test	Observed value	Reference range
White blood cell count (×10³/µL)	6.8	4.0-11.0
Neutrophils (%)	78	40-70
Lymphocytes (%)	14	20-40
Estimated absolute lymphocyte count (×10³/µL)*	0.95	1.0-4.0
Hemoglobin (g/dL)	11.2	13.5-17.5
Hematocrit (%)	34.1	41-53
Mean corpuscular volume (fL)	78	80-100
Platelets (×10³/µL)	412	150-400
Erythrocyte sedimentation rate (mm/hour)	72	<20
C-reactive protein (mg/dL)	8.6	<1.0
Total protein (g/dL)	9.1	6.0-8.3
Albumin (g/dL)	3.2	3.5-5.0
Aspartate aminotransferase (U/L)	38	10-40
Alanine aminotransferase (U/L)	34	7-56
HIV antigen/antibody	Nonreactive	Nonreactive
Respiratory viral polymerase chain reaction panel	Negative	Negative
Rapid group A streptococcal antigen	Negative	Negative
QuantiFERON-TB Gold	Negative	Negative

**Figure 1:**
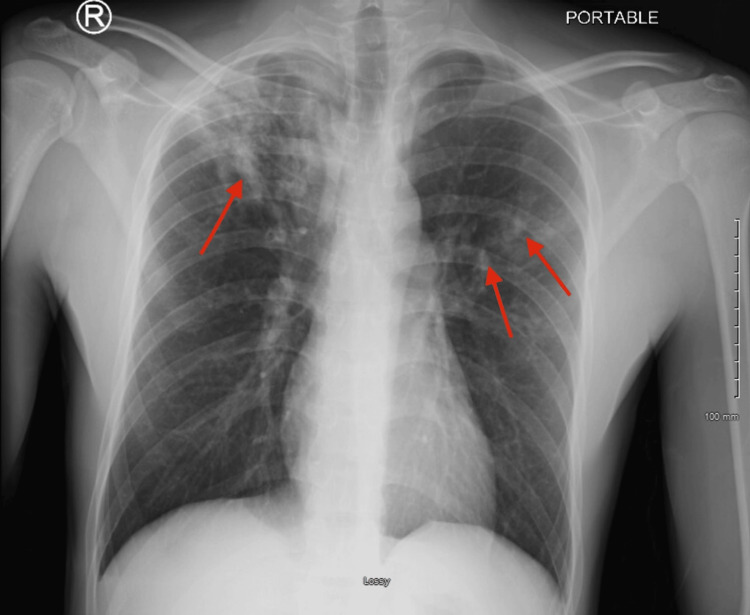
Chest radiograph demonstrating multifocal pulmonary opacities. A frontal chest radiograph demonstrates right upper-lobe airspace opacity and additional nodular opacities in the left upper and mid-lung fields (red arrows). These findings raised concern for multifocal infectious or granulomatous disease and prompted CT evaluation and airborne isolation. CT: computed tomography.

**Figure 2:**
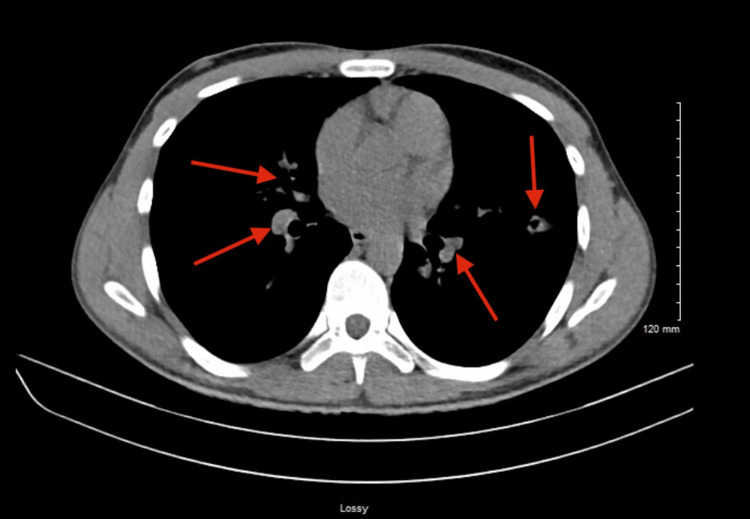
Non-contrast chest computed tomography (CT) demonstrating multilobar cavitary disease. Selected axial lung-window CT image demonstrates multiple air-containing cavities within areas of abnormal lung parenchyma, predominantly in the right upper lobe, with surrounding nodular consolidation (red arrows). Additional cavitary disease was present in the left upper and lower lobes on adjacent images. CT: computed tomography.

**Figure 3:**
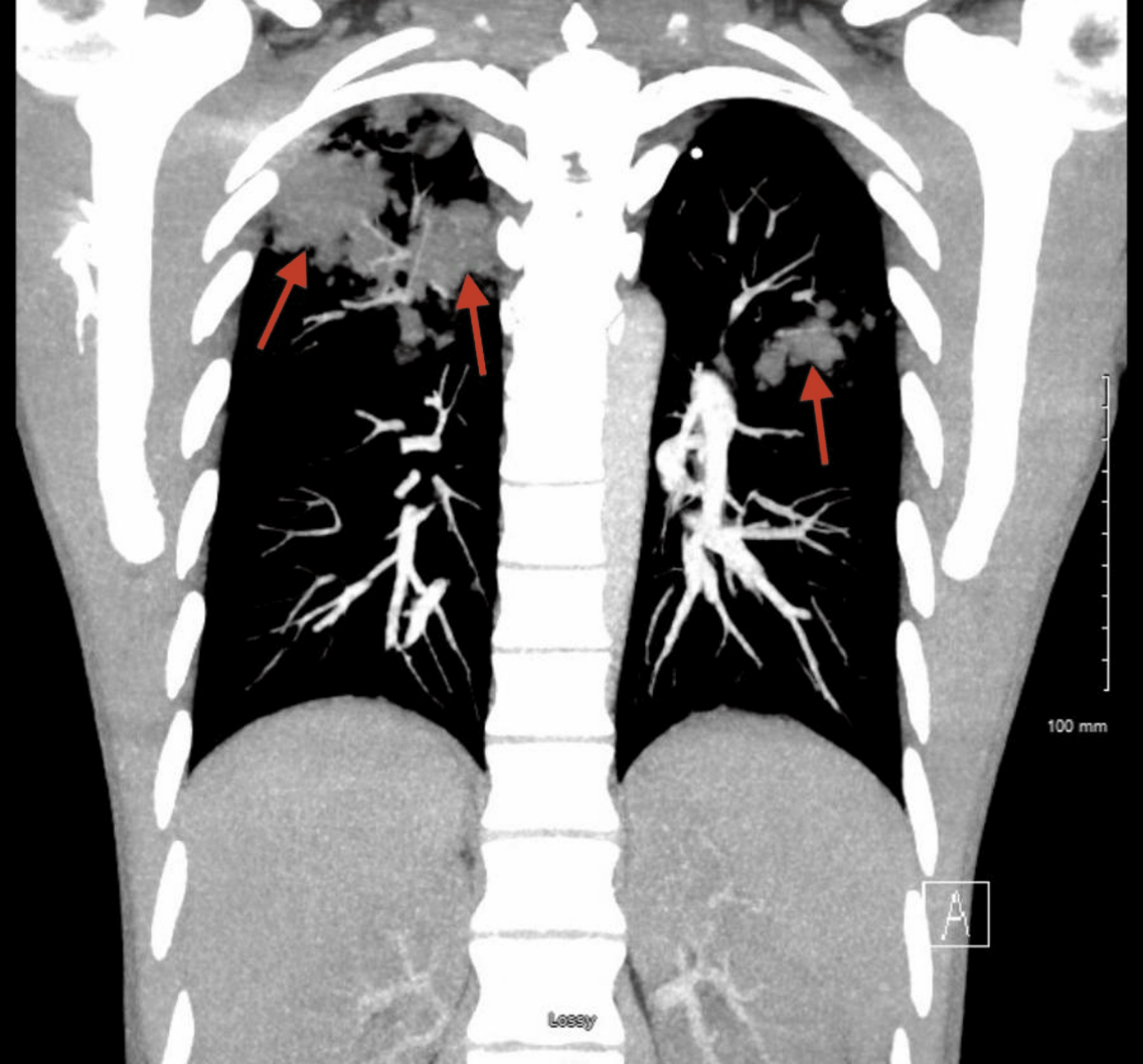
Contrast-enhanced chest CT demonstrating persistent cavitary and nodular disease. Selected axial contrast-enhanced CT image demonstrates persistent upper-lobe cavities and surrounding nodular consolidation (red arrows). In conjunction with positive AFB smears, positive *M. tuberculosis* NAAT, and subsequent culture growth, the findings supported active pulmonary TB. CT: computed tomography; AFB: acid-fast bacilli; NAAT: nucleic acid amplification testing; TB: tuberculosis; *M. tuberculosis*: *Mycobacterium tuberculosis*.

Chest radiography demonstrated right upper-lobe airspace opacity with additional nodular opacities in the left upper and mid-lung fields (Figure 1). Non-contrast computed tomography (CT) of the chest demonstrated numerous air-containing cavitary lesions, representing areas of parenchymal destruction, predominantly in the right upper lobe. Additional cavities involved the left upper lobe and left lower lobe, with adjacent nodular consolidation (Figure 2). Follow-up contrast-enhanced CT showed persistent multilobar cavitary lesions and scattered pulmonary nodules (Figure 3).

Because clinical and radiographic suspicion remained high despite the negative QuantiFERON result, the patient was placed in an airborne infection isolation room on hospital day 1. Sputum specimens were collected for AFB smear microscopy, *M. tuberculosis* NAAT, and mycobacterial culture. Two sputum smears were positive, the NAAT detected *M. tuberculosis*, and the final culture grew *M. tuberculosis* complex (Table 2). The culture result reflects final organism confirmation after mycobacterial incubation and was not available on the same day as specimen collection.

**Table 2:** Tuberculosis-directed microbiologic testing. AFB: acid-fast bacilli; *M. tuberculosis*: *Mycobacterium tuberculosis*.

Time point	Test	Result
Hospital day 1	Sputum AFB smear, sample 1	Positive, 3+
Hospital day 1	Sputum AFB smear, sample 2	Positive, 2+
Hospital day 2	*Mycobacterium tuberculosis* nucleic acid amplification test	Positive
Final culture result	Mycobacterial culture	*M. Tuberculosis* complex isolated

On hospital day 3, the patient began oral rifampin 600 mg daily, oral isoniazid 300 mg daily with oral pyridoxine 50 mg daily, oral pyrazinamide 1,500 mg daily, and oral ethambutol 1,200 mg daily. Baseline hepatic enzymes were reviewed before treatment. The patient received counseling regarding adherence, hepatotoxicity, ethambutol-associated visual symptoms, drug interactions, and transmission precautions.

The local health department was notified for contact investigation, DOT coordination, and isolation planning. During hospitalization, the fever curve improved, tachycardia resolved, hemoptysis did not recur, and the patient remained stable on room air. His cough and night sweats began to improve, and no early medication-limiting adverse effect was documented. Because sputum conversion and radiographic improvement generally require longitudinal assessment, outpatient sputum monitoring and interval chest imaging were arranged through the health department and infectious disease follow-up. Long-term sputum conversion, radiographic, and treatment-completion data were not available at the time of manuscript preparation. The clinical timeline is summarized in Table 3.

**Table 3:** Clinical timeline from symptom onset to diagnosis and treatment. AFB: acid-fast bacilli; CT: computed tomography; DOT: directly observed therapy; NAAT: nucleic acid amplification test; TB: tuberculosis.

Time point	Clinical event
Seven months before the presentation	Chronic cough began with intermittent hemoptysis
One month before the presentation	Progressive night sweats and fatigue
One week before the presentation	Acute worsening with fever, sore throat, and productive cough
Hospital day 0	Emergency department evaluation; abnormal chest radiograph
Hospital day 1	CT demonstrated multilobar cavitary disease; airborne isolation initiated; sputum collected
Hospital day 2	QuantiFERON-TB Gold negative; *M. tuberculosis* NAAT positive
Hospital day 3	AFB smear results reviewed; four-drug therapy initiated; health department notified
During hospitalization	Fever and tachycardia improved; no recurrent hemoptysis; stable oxygenation
Discharge and follow-up	DOT, home isolation instructions, contact investigation, sputum monitoring, and interval imaging arranged

## Discussion

This case illustrates two clinically important diagnostic pitfalls: a false-negative IGRA in microbiologically confirmed active TB and an atypical multilobar cavitary distribution. QuantiFERON-TB Gold measures a host immune response to TB-specific antigens; it does not directly detect organisms or establish whether disease is active. Therefore, a negative result should not override a high pretest probability based on symptoms, epidemiologic risk, and imaging [6].

False-negative IGRA results in active TB are not exceptional. In a multicenter cohort of 1,264 adults with culture-confirmed TB, Kwon et al. reported negative QuantiFERON-TB Gold In-Tube results in 182 patients (14.4%) [7]. Bilateral radiographic disease and lymphocytopenia were among the factors associated with false-negative results. Cho et al. likewise found that false-negative or indeterminate results occurred in active TB and were more frequent in patients with impaired immune responses or severe illness [8]. The present patient was young and had no recognized major immunosuppressive disorder, but he had bilateral multilobar disease, mild hypoalbuminemia, and an estimated absolute lymphocyte count of approximately 0.95 × 10³/µL. These findings may have reduced assay sensitivity, although the precise cause of his negative result cannot be established from a single case.

The patient’s epidemiologic background was also important. He was born in Venezuela and had a prolonged cough, intermittent hemoptysis, night sweats, and a family history of treated TB. This combination maintained a high clinical probability despite the negative IGRA. Current diagnostic guidance recommends respiratory sampling with AFB microscopy, NAAT, and culture when pulmonary TB is suspected [6,9]. AFB smear microscopy provides rapid evidence of mycobacteria but cannot distinguish *M. tuberculosis* from nontuberculous mycobacteria. NAAT rapidly supports identification of *M. tuberculosis*, while culture remains the microbiologic reference standard and permits drug-susceptibility testing.

The radiographic pattern further complicated recognition. Cavities in post-primary TB usually predominate in the upper lobes, but adult pulmonary TB has a broad imaging spectrum, including lower-lobe and multilobar disease [4,5]. This patient’s involvement of both upper lobes and the left lower lobe appropriately broadened the differential to include necrotizing bacterial pneumonia, fungal infection, septic emboli, vasculitis, and malignancy. The chronic symptom trajectory, epidemiologic risk, smear positivity, positive NAAT, and final culture result established TB despite the atypical distribution.

Quantitative interpretation of the laboratory data also supported chronic inflammatory disease without being specific for TB. The C-reactive protein level was approximately 8.6 times the upper reference limit, and the erythrocyte sedimentation rate was more than three times the upper limit. Mild microcytic anemia, thrombocytosis, hypoalbuminemia, and an increased globulin fraction were consistent with sustained inflammation. None of these abnormalities can diagnose TB, but together, they reinforced the need to investigate the prolonged systemic and respiratory syndrome rather than attribute it to an uncomplicated acute viral or bacterial upper respiratory infection.

The public health response was integral to management. Smear-positive cavitary pulmonary TB is associated with substantial transmission risk [4]. Airborne isolation, rapid NAAT, health department notification, contact investigation, and DOT coordination were therefore not ancillary measures but core components of care [2,3,9]. The patient demonstrated early symptomatic improvement after four-drug therapy, but microbiologic conversion and radiographic healing require follow-up over weeks to months and were not available for this report.

This report has limitations. It describes a single patient and cannot estimate the prevalence of false-negative IGRA results or atypical cavitary patterns. The exact QuantiFERON quantitative values and preanalytic handling details were unavailable, preventing assessment of whether a technical factor contributed. Bacille Calmette-Guérin vaccination status was unknown, although vaccination would not explain a false-negative IGRA. Drug-susceptibility results, long-term sputum conversion, interval radiographic findings, and final treatment-completion outcomes were not available at the time of manuscript preparation. Accordingly, the case should be interpreted as an educational illustration rather than evidence that all patients with negative IGRAs require treatment.

## Conclusions

A negative QuantiFERON-TB Gold assay does not exclude active pulmonary TB, even in a patient without recognized major immunosuppression. When prolonged constitutional and respiratory symptoms, epidemiologic risk, and cavitary imaging raise concern for TB, clinicians should maintain airborne precautions and pursue AFB smear microscopy, *M. tuberculosis* NAAT, and mycobacterial culture regardless of the IGRA result. This single case also illustrates that multilobar and lower-lobe cavitation may occur and can obscure an otherwise treatable and transmissible diagnosis. Prompt microbiologic confirmation, four-drug therapy, public health notification, contact investigation, DOT, and longitudinal sputum and imaging follow-up remain essential.
